# CGAL: computing genome assembly likelihoods

**DOI:** 10.1186/gb-2013-14-1-r8

**Published:** 2013-01-29

**Authors:** Atif Rahman, Lior Pachter

**Affiliations:** 1 Department of Electrical Engineering and Computer Sciences, 387 Soda Hall, UC Berkeley, Berkeley, CA 94720, USA; 2 Departments of Mathematics and Molecular & Cell Biology, 970 Evans Hall, UC Berkeley, Berkeley, CA 94720, USA

**Keywords:** Genome assembly, evaluation, likelihood, sequencing.

## Abstract

Assembly algorithms have been extensively benchmarked using simulated data so that results can be compared to ground truth. However, in *de novo *assembly, only crude metrics such as contig number and size are typically used to evaluate assembly quality. We present CGAL, a novel likelihood-based approach to assembly assessment in the absence of a ground truth. We show that likelihood is more accurate than other metrics currently used for evaluating assemblies, and describe its application to the optimization and comparison of assembly algorithms. Our methods are implemented in software that is freely available at http://bio.math.berkeley.edu/cgal/.

## Background

Genome assembly is the process of merging fragments of a DNA sequence produced by shotgun sequencing in order to reconstruct the original genome. The assembly problem is known to be NP hard for a number of formulations [1-3] and is also complicated by the many types of sequencing errors, experimental biases and the volume of data that must be processed. For these reasons, in addition to differences in underlying theory and algorithms, popular assembly methods employ many different heuristics and assemblies produced by existing methods differ substantially from each other [4,5].

Paradoxically, the difficulties of sequence assembly have been compounded by sequencing advances in recent years collectively termed next-generation sequencing technologies. Next-generation sequencing technologies such as 454 pyrosequencing by Applied Sciences [6], Solexa/Illumina sequencing, the SOLiD technology from Applied Biosystems and Helicos single-molecule sequencing [7] produce data of much greater volume at a much lower cost than traditional Sanger sequencing [8]. However, read lengths are considerably shorter and error rates are higher than those in Sanger sequencing. To allow *de novo *sequencing from short reads from next-generation sequencing machines several assemblers have been developed such as Velvet [9], Euler-sr [10], ABySS [11], Edena [12], SSAKE [13], VCAKE [14], SHARCGS [15], ALLPATHS [16], SOAPdenovo [17], Celera WGA [18], the CLC bio assembler and others [4,5]. A key problem that has arisen is to determine which assembler is 'the best'. In the past this has been done with the help of a number of measures such as N50 scaffold or contig lengths - which is the maximum contig (scaffold) length such that at least half the total length is contained in contigs (scaffolds) of length greater than or equal to that length. Although simulation studies show that simple metrics correlate with assembly quality, the currently used metrics are crude and provide only condensed summaries of the result. They can therefore be very misleading [5,19]. For example, the assembly consisting of simply gluing all reads end-to-end has a very large N50 length, but is obviously a poor assembly. Phillippy *et al*. presented software called amosvalidate [20] that identifies mis-assembly features and suspicious regions; however, it does not have high specificity and has not been widely adopted. Narzisi *et al*. used a feature-response curve [21] to rank assemblies based on features identified by amosvalidate. Studies such as [22-25] have discussed these issues and produce interesting insights into assembler performance but do not provide an intrinsic direct measure of assembly quality. The recent Assemblathon 1 competition used 10 different metrics [4] in an attempt to reveal more information than just N50 values, but most of the metrics can only be computed when the genome that is being assembled is known, and are therefore not useful in practice on real data.

In this paper we present a computationally efficient approach for computing the likelihood of an assembly, which provides a way to assess assemblies without a ground truth. Intuitively, the likelihood assessment evaluates the uniformity of coverage of the assembly, taking into account errors in the reads, the insert size distribution and the extent of unassembled data. Genome assembly by maximizing likelihood has been proposed previously by Myers [26] and Medvedev and Brudno [1] but their formulations are based on simplified models that do not use important parameters, especially the sequencing error. To demonstrate the power of our approach for assembly quality evaluation, we have implemented our methods in a program called CGAL. We have evaluated assemblies by testing several of them from different programs with varying input parameters in a setting where the desired target genome is known. For each assembly, we compute the likelihood using our tool and then compare our likelihood computation to standard measures such as N50 contig values, sequence similarity with the reference genome as well as values reported by amosvalidate. Although it is beyond the scope of this paper to compare all assemblers and explore all parameters, our results indicate that likelihood is meaningful and useful for evaluating assemblies.

## Results

Our overall approach is simple: we describe a probabilistic generative model for sequencing that captures many aspects of sequencing experiments, and from which we can compute the likelihood of an assembly. This intuitive framework is, however, complicated by one major difficulty, which is the problem we address in this paper: to compute the likelihood of an assembly it is necessary, in principle, to consider the possibility that a read was produced from every single location in the assembly. This results in an intractable computation, which we circumvent by approximating the likelihood via a reduction to a small set of 'likely' sites from which each read originated (using a mapping of the reads to the assembly). This requires an examination of the quality of the approximation, and leads to yet another difficulty, which is how to compute the likelihood for reads that do not map to the assembly at all. These issues are addressed in this paper and their solution is what enables our program for likelihood computation to be efficient and practical.

We begin by describing the statistical model that forms the basis for our likelihood computation. We believe that our model incorporates many aspects of typical sequencing experiments, but it can be easily generalized to accommodate additional parameters if desired.

### A generative model for sequencing

Let R = {*r*_1_, *r*_2_,..., *r_N_*} be a set of *N *paired-end (PE) (or mate pair) reads generated from a genome,  G (our model can, in principle, be adapted to single-end reads but we do not consider these here). We assume a fragment represented by two paired-end reads *r_i _*= (*r*_*i*1_, *r*_*i*2_) is generated according to the following model:

• A fragment length *l_i _*is selected according to a distribution *F*.

• A site for the 5' end of the fragment *s_i _*is selected according to a distribution *S*.

• The ends of the fragment are read as *r*_*i*1 _and *r*_*i*2 _according to an error model *E *which comprises mismatches as well as indels.

**Figure 1:**
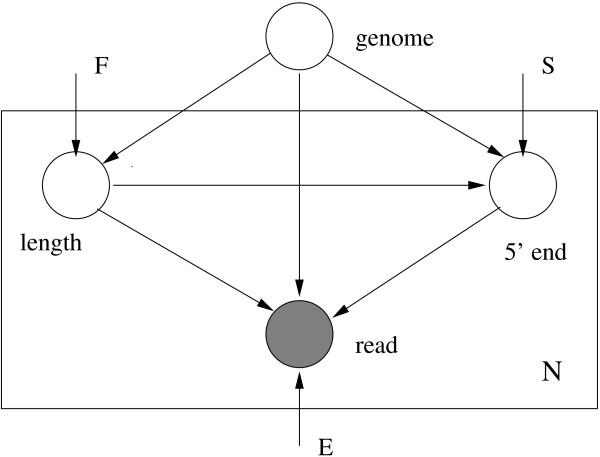
**A generative graphical model for sequencing**. *N *paired-end reads are generated independently from a genome. Here, *F *denotes the distribution of fragment lengths, *S *is the distribution of start sites of reads and *E *stands for error parameters.

The generative model is illustrated in Figure 1.

### Computing likelihood

Computing the likelihood of an assembly means that the probability of the (observed) set of reads is computed with respect to a proposed assembly using the model described in the previous section. The probability of a sequence of length *L *generating a paired-end or mate pair read (termed 'read' from now on) *r_i _*is

$$p\left(r_{i}\right)=\overset{L}{\underset{l=1}{\sum }}p_{F}\left(l\right)\overset{L-l+1}{\underset{s=1}{\sum }}p_{S}\left(s\right)\underset{e\in E}{\sum }p_{E}\left(r_{i}|a_{s}\ldots a_{s+l-1},e\right)$$

where *a_s _*... *a*_*s *+ l - 1 _is the assembly subsequence starting at *s *of length *l, E *denotes all possible ways of obtaining *r_i _*from *a_s _*... *a*_*s *+ l - 1_. *p_F _*(*l*) is the probability that the fragment length is *l, p_S_*(*s*) is the probability that the 5' end of the fragment is at site *s *and *p_E_*(*r_i_*|*a_s _*... *a*_*s *+ l - 1_, *e*) is the probability of obtaining *r_i _*from *a_s _*... *a*_*s *+ l - 1 _with sequencing errors given by *e*.

Although in theory a read could have been generated from any site (assuming that every base could have been an error), in practice the probability decreases considerably with increasing number of disagreements between the source sequence and the read sequence. We therefore approximate the probability *p*(*r_i_*) by mapping the read to the assembly and ignoring mappings with a large number of differences. If *M_i _*is the number of such mappings of read *r_i_*, the probability is given by

$$p\left(r_{i}\right)\approx \overset{M_{i}}{\underset{j=1}{\sum }}p_{F}\left(l_{i,j}\right)p_{S}\left(s_{i,j}\right)p_{E}\left(r_{i}|a_{i,j},e_{i,j}\right)$$

where *l_i, j_, s_i, j_, a*_*i, j *_and *e*_*i, j *_are the fragment length, start site, assembly subsequence and errors corresponding to the *j*th mapping of the *i*th read, respectively. The above equation generalizes to assemblies with more than one contig. Given an assembly  Aand a set of reads R = {*r*_1_, *r*_2_,..., *r_N_*}, the log likelihood is given by

$$\begin{array}{ccc} l\left(A;R\right) & =\text{log}\overset{N}{\underset{i=1}{\prod }}p\left(r_{i}|A\right) & \\ & \approx \overset{N}{\underset{i=1}{\sum }}\text{log}\overset{M_{i}}{\underset{j=1}{\sum }}p_{F}\left(l_{i,j}\right)p_{S}\left(s_{i,j}\right)p_{E}\left(r_{i}|a_{i,j},e_{i,j}\right). \end{array}$$

In the above equation *M_i _*≥ 1 for all reads *r_i_*, and in Methods we explain how we obtain alignments for all reads and how to learn the needed distributions.

### Validation with simulated data

To test our implementation, we developed a simulator that generates reads according to given error parameters and fragment lengths distributed according to a Gaussian distribution.

We generated 3 million 35 bp paired-end reads from a strain of *Escherichia coli *([NCBI: NC_000913.2]) and an assembly of *Grosmannia clavigera *([DDBJ/EMBL/GenBank: ACXQ00000000]) reported in [27]. Table 1 shows the percentage difference in likelihood values computed using true parameters provided to the simulator and using parameters inferred by CGAL.

**Table 1:** Percentage difference between the simulator and CGAL

Genome	Length (bp)	Percentage difference
*E. coli*	4.6 M	0.074
*G. clavigera*	29.1 M	0.0755

bp, base pair.

### Performance of assemblers on *E. coli *reads

**Figure 2:**
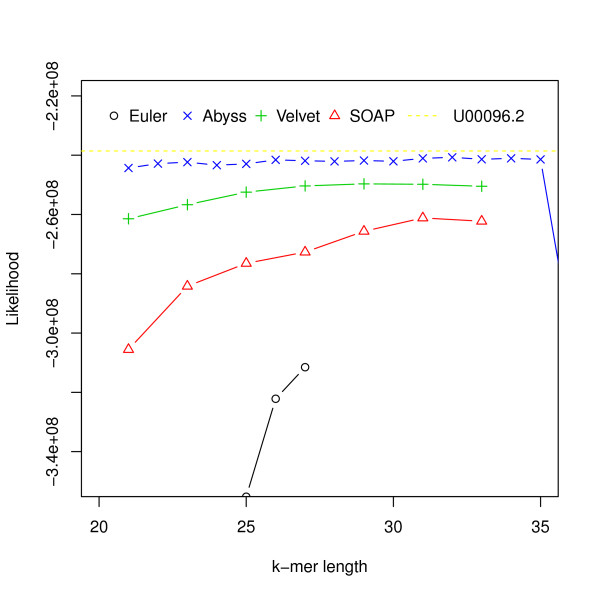
**Hash length vs log likelihood for *E. coli***. Log likelihoods of assemblies of *E. coli *reads are shown on the *y*-axis. Assemblies are generated using different assemblers for varying k-mer length, which is shown on the *x*-axis. The dotted line corresponds to the log likelihood of the reference.

We assessed the performance of four assemblers: Velvet, Euler-sr, ABySS and SOAPdenovo on an *Escherichia coli *dataset ([SRA:SRR 001665] and [SRA:SRR 001666]). We chose *E. coli *because its assembly is a true 'gold standard' without questions about reliability or accuracy. We assembled the reads using the assemblers mentioned for different hash lengths (k-mer was used for constructing the de Bruijn graph [10]). Likelihood values for assemblies along with the likelihood value for the reference ([NCBI: U00096.2]) are shown in Figure 2.

**Figure 3:**
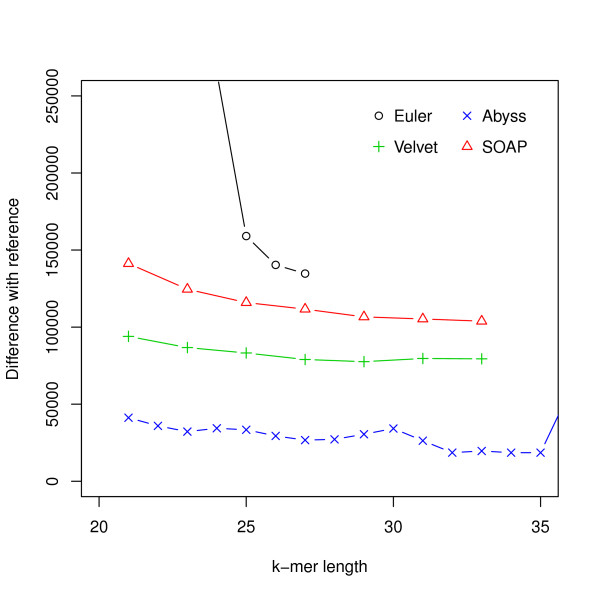
**Hash length vs difference from reference for *E. coli***. The differences between assemblies and the reference are shown on the *y*-axis where the difference refers to the numbers of bases in the reference not covered by the assembly or differ between the reference and the assembly.

**Figure 4:**
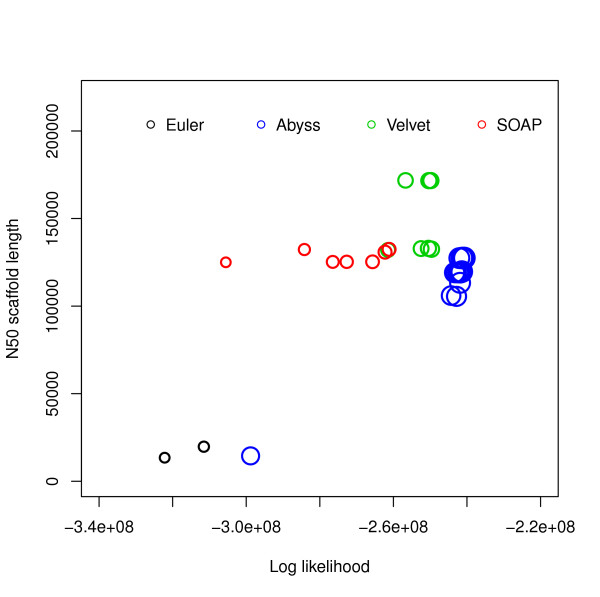
**Log likelihood vs N50 scaffold length for *E. coli***. Log likelihoods are shown on the *x*-axis and N50 scaffold lengths are shown on the *y*-axis. Each circle corresponds to an assembly generated using an assembler for some hash length and the sizes of the circles correspond to similarity with reference. The *R*^2 ^values are: (i) log likelihood vs similarity: 0.9372048, (ii) log likelihood vs N50 scaffold length: 0.44011, (iii) N50 scaffold length vs similarity: 0.3216882.

**Figure 5:**
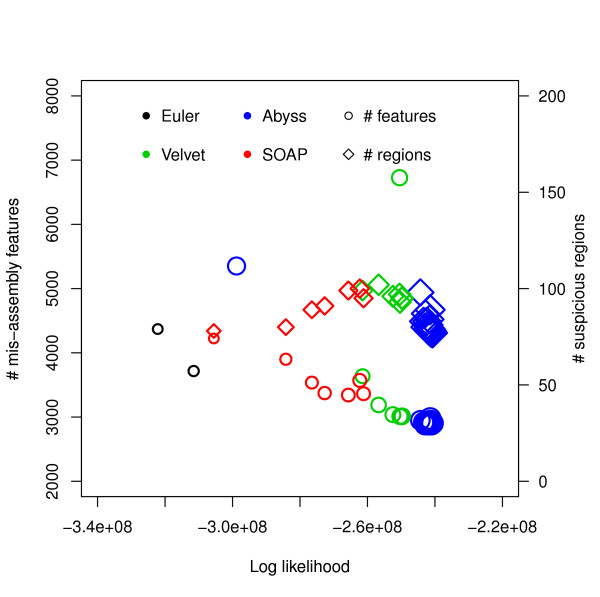
**Log likelihood vs numbers of mis-assembly features and suspicious regions for *E. coli***. Log likelihoods are shown on the *x*-axis and numbers of mis-assembly features and suspicious regions reported by amosvalidate are shown on the *y*-axis. Each symbol corresponds to an assembly generated using an assembler for some hash length and the sizes of the symbols correspond to similarity with reference. The *R*^2 ^values are: (i) log likelihood vs number of mis-assembly features: 0.8922, (ii) log likelihood vs number of suspicious regions: 0.9039, (iii) similarity vs number of mis-assembly features: 0.8211, (iv) similarity vs number of suspicious regions: 0.7723.

For this dataset ABySS outperforms the others when likelihood is used as the metric. We also aligned the assemblies to the reference with NUCmer [28] and Figure 3 shows the differences from the reference against the hash lengths. The relations among likelihood, N50 length and similarity are illustrated in Figure 4 and Additional file 1, Figure S1. They suggest that likelihood values are better at capturing sequence similarity than other metrics commonly used for evaluating assemblies, such as the N50 scaffold or contig lengths. We also ran the amosvalidate pipeline to obtain the numbers of mis-assembly of features and suspicious regions (Figure 5) and plotted the feature response curves (FRCs) [21] of the assemblies (Additional file 1, Figures S4, S5). The FRCs also rank an ABySS assembly as the best one.

**Figure 6:**
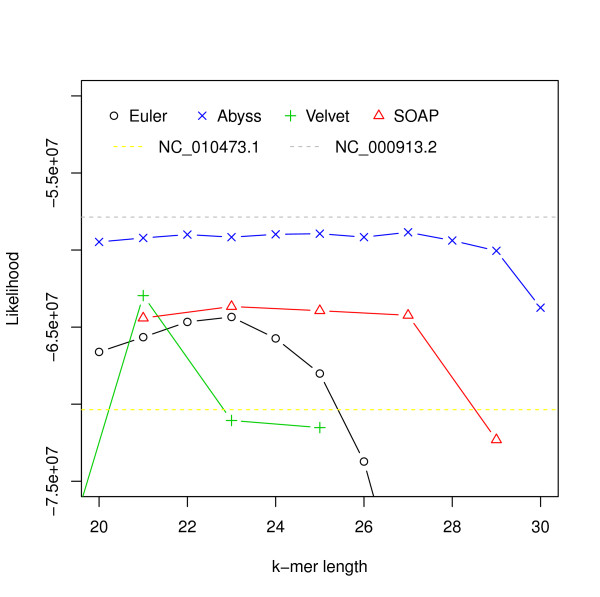
**Hash length vs log likelihood for *E. coli *data from CLC bio**. Log likelihoods of assemblies of *E. coli *reads from CLC bio are shown on the *y*-axis. Assemblies are generated using different assemblers for varying k-mer length, which is shown on the *x*-axis. The yellow dotted line corresponds to the log likelihood of the reference provided and the gray dotted line corresponds to the log likelihood of the strain we believe the reads were generated from.

A similar analysis was performed on a different *Escherichia coli *dataset downloaded from CLC bio [29]. It consists of approximately 2.6 million 35 bp paired-end Illumina reads (approximately 40 times coverage) along with a reference genome ([NCBI: NC_010473.1]). We noticed that many of the assemblies have a better likelihood than the reference. However, we assembled reads that could not be mapped to the reference and after running BLAST [30] we found another substrain of *Escherichia coli *strain K-12, MG1655 ([NCBI: NC_000913.2]), which has a better likelihood than all assemblies. We conjecture that the reads were generated from NC_000913.2. Likelihood values are shown in Figure 6 and relationships among likelihood, similarity and N50 values are illustrated in Additional file 1, Figures S6-S10.

### Performance of assemblers on *G. clavigera *reads

To assess assemblies of a larger genome, we used the dataset generated for sequencing an ascomycete fungus, *Grosmannia clavigera *by DiGuistini *et al. *[27]. We ran Velvet, ABySS and SOAP on PE Illumina reads with a mean fragment length of 200 bp [SRA:SRR 018008-11] and 700 bp [SRA:SRR 018012].

**Figure 7:**
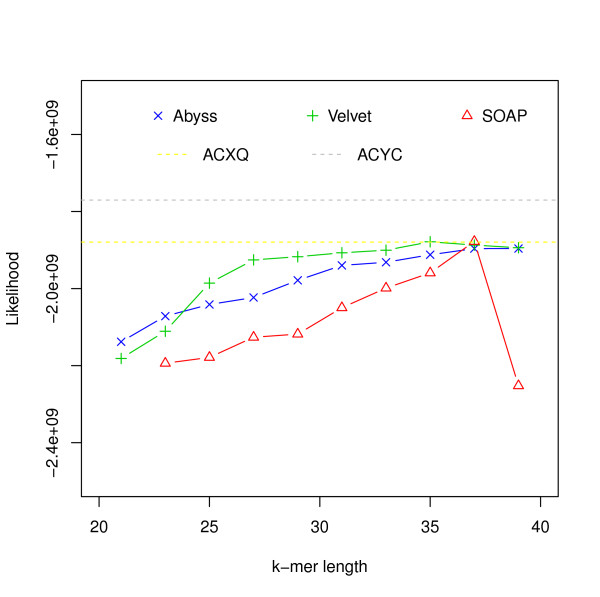
**Hash length vs log likelihood for *G. clavigera***. Log likelihoods of assemblies of *G. clavigera *reads are shown on the *y*-axis. Assemblies are generated using different assemblers for varying k-mer length, which is shown on the *x*-axis. The dotted lines correspond to the log likelihoods of the assemblies generated using Sanger, 454 and Illumina data.

The likelihood values of the 200 bp fragment reads for the assemblies are shown in Figure 7. It also shows likelihood values for assemblies [DDBJ/EMBL/GenBank: ACXQ00000000] and [DDBJ/EMBL/GenBank: ACYC000 00000] reported in [27], which were generated using Sanger and 454 reads as well as Illumina reads. The numbers of mis-assembly features and suspicious regions identified by amosvalidate and the feature response curves are shown in Additional file 1, Figures S14-S15.

**Figure 8:**
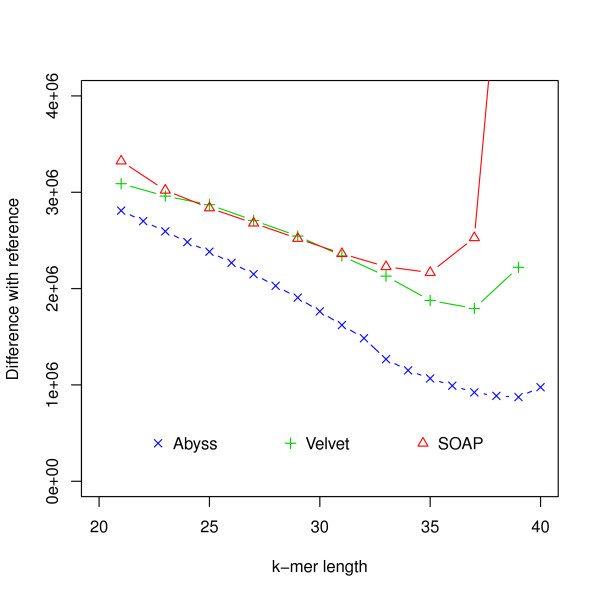
**Hash length vs difference from reference for *G. clavigera***. Differences between assemblies and the reference are shown on the *y*-axis where difference refers to the numbers of bases in the reference not covered by the assembly or differ between the reference and the assembly.

**Figure 9:**
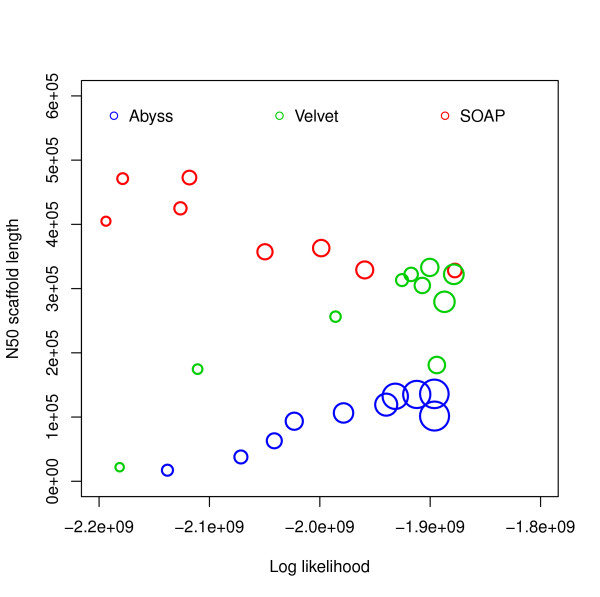
**Log likelihood vs N50 scaffold length for *G. clavigera***. Log likelihoods are shown on the *x*-axis and N50 scaffold lengths are shown on the *y*-axis. Each circle corresponds to an assembly generated using an assembler for some hash length and the sizes of the circles correspond to similarity with reference. The *R*^2 ^values are: (i) log likelihood vs similarity: 0.4545793, (ii) log likelihood vs N50 scaffold length: 0.002397233, (iii) N50 scaffold length vs similarity: 0.006084032.

Figure 8 shows that the assembly with most sequence coverage is produced by ABySS. However, in this case ABySS assemblies are much longer compared to other assemblies and references (Additional file 1, Tables S9-S11). This results in lower likelihoods compared to some assemblies by Velvet and SOAPdenovo. Figure 9 and Additional file 1, Figure S11 show relationships among likelihood, similarity and N50 values. In FRC analysis, genome coverage is estimated using assembly length and so it does not take into account the unassembled sequences and ranks ABySS assemblies above others. It is interesting that assemblies with the best likelihood and sequence similarity are generated for higher values of hash length than are optimal for producing high N50 values.

### GAGE results

We computed likelihoods for the assemblies generated in the GAGE project [5]. In Additional file 1, Tables S12-S14 show likelihoods of Library 1 and the number of reads mapped to assemblies by Bowtie 2 [31]. We found that the likelihood values of Library 1 are dominated by coverage and contiguity does not affect these values greatly. However, contiguity has more effect on the likelihoods of Library 2, which has a longer insert size (Additional file 1, Tables S12-S14), as might be expected. The total likelihood along with coverage and N50 values are shown in Tables 2, 3, 4, 5. For human chromosome 14, we computed Library 2 likelihoods for assemblies with the best three likelihoods of Library 1. The likelihood values of Library 2 for bumble bee assemblies were not computed as only a small fraction of the reads could be mapped to the assemblies.

**Table 2:** Likelihoods of GAGE assemblies of *S. aureus*

Assembler	Likelihood	Number of reads mapped	Coverage (%)	Scaffold N50 (kb)	Contig N50 (kb)
ABySS	-23.34 × 10^7^	1236230	99.74^a^	34	29.2

ALLPATHS-LG	-24.53 × 10^7^	1220328	99.38	1092	96.7

Bambus2	-23.76 × 10^7^	1200527	98.68	1084	50.2

MSR-CA	-25.85 × 10^7^	1192001	98.70	2412	59.2

SGA	-26.61 × 10^7^	1018936	98.09	208	4.0

SOAPdenovo	-23.55 × 10^7^	1212384	99.62	332	288.2

Velvet	-23.28 × 10^7^	1203907	99.21	762	48.4

Reference	-22.38 × 10^7^	1268718	-	-	-

^a ^Value reported in original paper is 98.63.

**Table 3:** Likelihoods of GAGE assemblies of *R. sphaeroides*

Assembler	Likelihood	Number of reads mapped	Coverage (%)	Scaffold N50 (kb)	Contig N50 (kb)
ABySS	-27.55 × 10^7^	1199197	99.11^a^	9	5.9

ALLPATHS-LG	-26.61 × 10^7^	1237938	99.53	3192	42.5

Bambus2	-32.56 × 10^7^	1111596	95.07	2439	93.2

CABOG	-39.23 × 10^7^	1022732	92.49	66	20.2

MSR-CA	-31.61 × 10^7^	1155078	96.48	2976	22.1

SGA	-31.58 × 10^7^	1031547	97.69	51	4.5

SOAPdenovo	-27.67 × 10^7^	1212959	99.12	660	131.7

Velvet	-28.77 × 10^7^	1176125	98.40	353	15.7

Reference	-25.99 × 10^7^	1255750	-	-	-

^a ^Value reported in original paper is 96.99.

**Table 4:** Likelihoods of GAGE assemblies of human chromosome 14

Assembler	Likelihood	Number of reads mapped	Coverage (%)	Scaffold N50 (kb)	Contig N50 (kb)
ABySS	-23.44 × 10^8^	22096466	82.22	2.1	2

ALLPATHS-LG	-22.77 × 10^8^	23122569	97.24	81647	36.5

CABOG	-21.26 × 10^8^	23433424	98.32	393	45.3

SOAPdenovo	^a^	^a^	98.17	455	14.7

Reference	-19.04 × 10^8^	23978017	-	-	-

^a ^Likelihood not computed as reads could not be mapped with Bowtie 2.

**Table 5:** Likelihoods of GAGE assemblies of a bumblebee, *B. impatiens*

Assembler	Likelihood	# reads mapped	Scaffold N50 (kb)	Contig N50 (kb)
ABySS	-30.83 × 10^9^	72629126	-	-

CABOG	-19.99 × 10^9^	92844610	1125	23.5

MSR-CA	-22.84 × 10^9^	78755756	1246	32.4

SOAPdenovo	a	a	1374	57.1

^a ^Likelihood not computed as reads could not be mapped with Bowtie 2.

### Assemblathon 1 results

**Figure 10:**
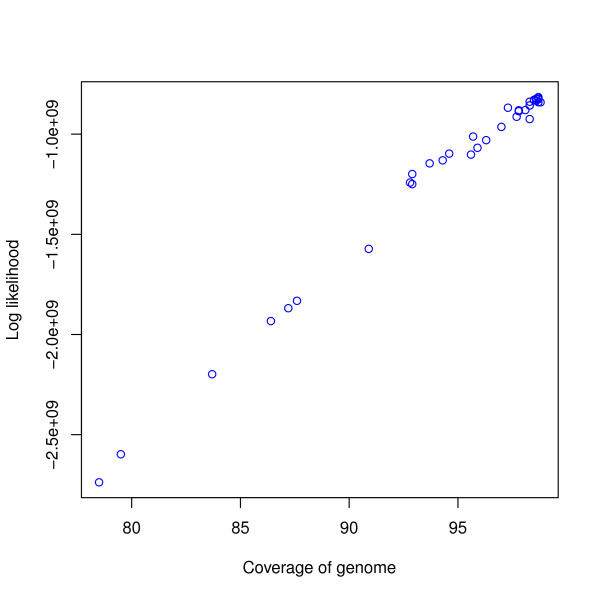
**Coverage vs log likelihood for Assemblathon 1 entries**. Coverage is shown on the *x*-axis and log likelihood is shown on the *y*-axis. Each circle corresponds to an assembly. The *R*^2 ^value is 0.989972.

We also analyzed the assemblies submitted for Assemblathon 1 [4]. The likelihoods for a library of an insert size of mean 200 bp for all assemblies are given in Additional file 1, Table S15. Figure 10 shows the relationship between likelihood and coverage. We took the entries with the highest likelihood for the top ten participants and computed the likelihoods for the libraries of insert sizes of means 3,000 bp and 10,000 bp. Table 6 shows the total likelihoods of the top ten participants along with their Assemblathon 1 rankings.

**Table 6:** Likelihoods of Assemblathon 1 assemblies

Assembler	Likelihood	Number of reads mapped	Assemblathon 1 rank
BGI 1	-20.17 × 10^8^	42005212	2

CSHL 2	-20.19 × 10^8^	41973576	5

BCCGSC 5	-20.23 × 10^8^	41891758	7

IoBUGA 2	-20.49 × 10^8^	41931526	9

RHUL 3	-20.69 × 10^8^	41753084	10

DOEJGI 1	-20.73 × 10^8^	41836210	4

WTSI-P 2	-20.81 × 10^8^	41748504	11

Broad 1	-21.75 × 10^8^	41778343	1

EBI 1	-21.83 × 10^8^	41377165	8

WTSI-S 4	-30.81 × 10^8^	37442672	3

## Discussion

### E. coli

We found that for both *E. coli *datasets, the assemblies with the best likelihoods were constructed by ABySS. They also have most similarity with the references (assuming [NCBI: NC 000913.2] is the reference for the CLC bio dataset). The *R*^2 ^values (Figures 4, 5 and Additional file 1, Figure S1) reveal that the likelihoods reflect sequence similarity better than contiguity statistics such as N50 values as well as the numbers of mis-assembly features and suspicious regions identified by amosvalidate. The analysis of the two different *E. coli *datasets also reveal that for assemblers like Velvet and SOAPdenovo higher likelihood values are achieved for different values of the k-mer length used to construct the de Bruijn graph during assembly.

### G. clavigera

For the *G. clavigera *dataset, one of the Velvet assemblies has the highest likelihood. Although ABySS assemblies have more coverage, they have lower likelihood because of the much longer total length. Despite this we see from the *R*^2 ^values that likelihood values reflect sequence similarity better than the N50 values (Figure 9 and Additional file 1, Figures S11, S14) and the numbers of mis-assembly features and suspicious regions reported by amosvalidate. This suggests that likelihood values are useful in simultaneously evaluating coverage and total assembly length.

### GAGE

For the GAGE *Staphylococcus aureus *dataset, we find that the assembly generated using Velvet has the best likelihood but likelihoods of a few other assemblies are close. For *Rhodobacter sphaeroides*, the ALLPATHS-LG assembly has the best likelihood, which is also the assembly with the highest coverage and N50 scaffold length. The CABOG assembly of human chromosome 14 is the one with the best likelihood. The CABOG assembly also has the highest coverage and N50 contig length among the assemblies. In all three cases, we find that the reference sequences have the highest likelihoods and the highest number of reads mapped to them by Bowtie 2. For the bumblebee data, the assembly using CABOG has the best likelihood of the three (the likelihood of the SOAPdenovo assembly could not be computed as reads could not be mapped to it using Bowtie 2).

### Assemblathon 1

Figure 10 reveals that for the Assemblathon 1 dataset, the likelihoods for a small fragment library correlate well with coverages. Overall, we find that participants with the ten highest likelihoods were ranked within the top eleven by the Assemblathon 1 organizers but there are differences between the two rankings. The entry with the highest likelihood is from the Beijing Genomics Institute (BGI), which was ranked two in the original paper. The differences in rankings are primarily due to the emphasis on contiguity made by the Assemblathon 1 organizers while our likelihood model implicitly places more importance on coverage. This brings up the issue that better contiguity statistics can be achieved by not reporting hard-to-assemble regions and these values may be misleading if they are not used in conjunction with an indicator of coverage.

### Applications

Currently, assembly evaluation projects rely mostly on simulated data or data from genomes that have been sequenced previously [4,5]. Having a tool that can assess the quality of assembly without the need for a reference will allow researchers who work with real data from genomes that have not been sequenced before to assess the performance of different assemblers on their data, and to optimize the parameters in the programs they are using.

The analysis of two different datasets from *E. coli *reveals that the performance of some assemblers varies significantly depending on the k-mer chosen for constructing the de Bruijn graph. Moreover, the 'optimal' value depends on read length and sequence coverage. Likelihood values can therefore guide selection of parameter values.

The concept of maximum likelihood genome assembly was introduced by Medvedev and Brudno [1] but they do not consider sequencing errors or paired-end reads. A likelihood model taking into account these may be the next step towards genome assemblers for real data that try to maximize likelihood.

## Conclusions

In this paper we presented a tool for computing the likelihood of an assembly. The result can be used as a metric for evaluating and comparing assemblies. In the past this has been done using many different criteria including N50 lengths, total sequence length and number of contigs. The likelihood model incorporates these directly or indirectly in addition to other important factors such as genome coverage and assembly accuracy and combines them into a single metric for evaluation.

We have also used our tool to assess the performance of assemblers using different datasets. Our results indicate that likelihood reflects sequence similarity, which is missed by other metrics commonly used and will be a valuable tool for evaluating assemblies generated by different assemblers and for different values of the input parameters.

## Materials and methods

### Mapping reads

The first step in computing the likelihood is mapping reads to the assembly. A number of tools are available for this such as Bowtie [31,32], MAQ [33], BWA [34] and BFAST [35]. Our present implementation can use either BFAST or Bowtie 2 for mapping reads as they support mapping with indels and report multiple alignments in a way that gives all the required information without accessing the assembly sequence. But any tool that reports multiple alignments of reads and allows for insertions and deletions can be used with some minor modifications.

However, existing tools do not usually map all reads, and for the likelihood computation it is necessary to assign probabilities to reads that are unmapped. We found that mapping tools were unable to map a large fraction of reads in our experiments. One option is to assign probabilities to these reads, assuming that they could have been generated from any site, using the number and types of errors not handled by the mapping tool. But it is then often the case that unmapped reads are deemed more probable than mapped ones, which we believe is anomalous. Furthermore, in our analyses we determined that the resulting probabilities were inaccurate (results not shown). Therefore, we chose to directly align the reads not mapped by BFAST or Bowtie 2 using an adaptation of the Smith-Waterman algorithm. We adapted the striped implementation of the Smith-Waterman algorithm by Farrar [36]. This step is time consuming, so we align only a random subset of reads with the number specified by the user and approximate probabilities using these.

### Learning distributions

To compute the likelihood from mapped reads, we need to learn the distribution of fragment lengths, their distribution across the genome and error characteristics. Since they differ with library preparation methods and sequencing instruments, we chose to learn these from sequencing data generated in the experiment. We do this by mapping reads to the assembly and using reads that map uniquely. However, this can be easily extended to take into account all reads by using the expectation-maximization (EM) algorithm at the expense of more iterations. We explain each distribution in more detail below.

#### Fragment length distribution

The distribution of fragment lengths depends on the method used for size selection and may not be approximated well by common distributions [37]. So, we use the empirical distribution.

#### Distribution of fragments along genome

In our implementation, we assume that fragments are distributed uniformly across the genome. We leave incorporating sequencing bias as future work.

#### Error model

In the error model used at present, we have made the assumption that sequencing errors are independent of one another. We learn an error rate for each position in the read since error rates are known to be different across positions in reads [38]. We also learn separate error rates for each type of base and substitution type. Although errors are known to depend on sequence context [38], we have ignored them for the sake of simplicity.

To account for varying indel rates across positions in reads, we learn an insertion rate and a deletion rate for each position in the read. Since short indels are more likely than longer ones, we also count the number of insertions and deletions by length.

### Implementation

As mentioned earlier, we use BFAST or Bowtie 2 to map reads to assemblies. The parameters are set so that they report all alignments of a read found.

The remaining code for computing likelihood is written in C++ and it consists of three parts:

**convert**: This converts the output generated by BFAST or Bowtie 2 to an internal format. It also separates reads with no end or one end mapped and reads with ends mapped to different scaffolds if needed. Separating this module also allow us to support other mapping tools by writing a conversion routine.

**align**: To align the reads not mapped by the mapping tool, we adapted the striped implementation of the Smith-Waterman algorithm by Farrar [36]. As this step is time consuming, we align a random subset of reads with the number determined by the user. This step is multithreaded to speed up the process.

**CGAL**: This learns the fragment length distribution and parameters for the error model using uniquely mapped reads and then uses these to compute the likelihood value.

### Assembling genomes

To assemble reads, we varied the k-mer length used to construct the de Bruijn graph to obtain different assemblies for each assembly tool. For other parameters, the default values or values suggested in manuals were used.

### Data analysis

Likelihoods were computed by running CGAL with the default parameters and aligning between 300 and 1,000 randomly chosen reads not mapped by the mapping tool used. The running time for CGAL was approximately 1/3 of the time taken to map reads using Bowtie 2.

To compute the difference between an assembly and the reference, we aligned the assembly to the reference using NUCmer [28]. The difference refers to the number of bases in the reference that are either not covered by the assembly or differ in the reference and assembly. Contigs were generated by splitting scaffolds at sites with 25 or more *N*'s (character representing any base).

## Abbreviations

bp: base pair; FRC: feature response curve; PE: paired-end.

## Competing interests

The authors have no competing interests.

## Authors' contributions

AR and LP conceived the project and developed the methodology. AR implemented the method in the CGAL software and obtained the results of the paper. AR and LP wrote the manuscript. All authors read and approved the final manuscript.

## Supplementary Material

Additional File 1 **Supplementary information for computing genome assembly likelihoods**. Additional figures, tables and information to supplement the text. Click here for file
