# Supplementary material for: CGAL: computing genome assembly likelihoods
Source: Genome Biol. 2013 Jan 29;14(1):R8. doi: 10.1186/gb-2013-14-1-r8 (PMC3663106; doi:10.1186/gb-2013-14-1-r8)
Supplement: Additional File 1 — Supplementary information for computing genome assembly likelihoods. Additional figures, tables and information to supplement the text. [file gb-2013-14-1-r8-S1.PDF]

Supplementary information for computing genome assembly likelihoods

Atif Rahman[1] , Lior Pachter[1,2*]

[1]Department of Electrical Engineering and Computer Sciences, 387 Soda Hall, UC Berkeley, Berkeley, CA 94720, USA
[2]Departments of Mathematics and Molecular & Cell Biology, 970 Evans Hall, UC Berkeley, Berkeley, CA 94720, USA

Email: Atif Rahman - atif@eecs.berkeley.edu; Lior Pachter*- lpachter@math.berkeley.edu;

*Corresponding author

The Assemblers

The following assemblers were used for assembling reads from *E. coli* and *G. clavigera*:

- ABySS-1.2.7
- Euler-sr.1.1.2
- SOAPdenovo-V1.05
- Velvet_1.1.04

Supplementary information for E. coli data

- Organism: *Escherichia coli*
- Total reads: 10408224
- Reads mapped using: Bowtie 2 - 2.0.0 - beta 6

Reference

- Identifier: [NCBI: U00096.2]
- Likelihood: -238572967.687551
- Number of reads mapped: 10305539
- Total length: 4639675 bases

Assemblies

k-mer	# contigs	Log likelihood	# reads mapped	Total length	N50 scaffold	N50 contig	Diff
21	775	-24.43×10^7	10239632	4716697	106014	106014	41232
22	577	-24.28×10^7	10252838	4655390	105565	105565	35940
23	466	-24.23×10^7	10260818	4668411	119098	113303	32206
24	423	-24.33×10^7	10258113	4662049	119103	119103	34380
25	385	-24.29×10^7	10260950	4647968	119103	119103	33392
26	296	-24.16×10^7	10267506	4776771	119289	119289	29411
27	290	-24.19×10^7	10270072	4655849	113303	113303	26690
28	268	-24.21×10^7	10269192	4653402	127397	127397	27186
29	251	-24.18×10^7	10267174	4655844	119060	119060	30500
30	243	-24.20×10^7	10262735	4648199	119061	119061	34214
31	200	-24.11×10^7	10273191	4660482	127380	127380	26299
32	183	-24.07×10^7	10278821	4720013	127449	127449	18566
33	191	-24.14×10^7	10274370	4715256	127449	127449	19629
34	184	-24.11×10^7	10275712	4677901	127449	127449	18549
35	187	-24.14×10^7	10271870	4678986	119691	114019	18551
36	1357	-29.88×10^7	9694443	4781873	14486	9005	58656

Table S1: Details of ABySS assemblies of *E. coli*

k-mer	# contigs	Log likelihood	# reads mapped	Total length	N50 scaffold	N50 contig	Diff
23	8721	-79.29×10^7	5450331	4173530	941	941	596466
24	3915	-50.08×10^7	7963793	4546541	2675	2675	265291
25	1556	-35.52×10^7	9294984	4714101	6828	6828	159086
26	818	-32.21×10^7	9686807	4867555	13445	13445	140369
27	567	-31.15×10^7	9777746	4627451	19731	19731	134725

Table S2: Details of Euler-sr assemblies of *E. coli*

k-mer	# contigs	Log likelihood	# reads mapped	Total length	N50 scaffold	N50 contig	Diff
21	103	-30.55×10^7	9769728	4536683	125011	42063	141257
23	108	-28.42×10^7	9894505	4547233	132296	47267	124586
25	126	-27.65×10^7	9947906	4553034	125245	58712	115939
27	142	-27.27×10^7	9989334	4555420	125292	63059	111662
29	133	-26.57×10^7	10033409	4556756	125296	60809	106641
31	131	-26.12×10^7	10055340	4561246	132417	59343	105305
33	137	-26.23×10^7	10057803	4558913	130824	63523	103877

Table S3: Details of SOAPdenovo assemblies of *E. coli*

k-mer	# contigs	Log likelihood	# reads mapped	Total length	N50 scaffold	N50 contig	Diff
21	146	-26.14×10^7	10073984	4558369	132072	39884	94001
23	152	-25.67×10^7	10114372	4558963	171787	47058	86735
25	148	-25.25×10^7	10153953	4561882	132844	50967	83227
27	137	-25.04×10^7	10176741	4561981	171642	60809	78998
29	138	-24.97×10^7	10180739	4564097	132509	64114	77588
31	137	-24.98×10^7	10183271	4564728	171679	64138	79649
33	143	-25.05×10^7	10174263	4568906	132976	39335	79423

Table S4: Details of Velvet assemblies of *E. coli*

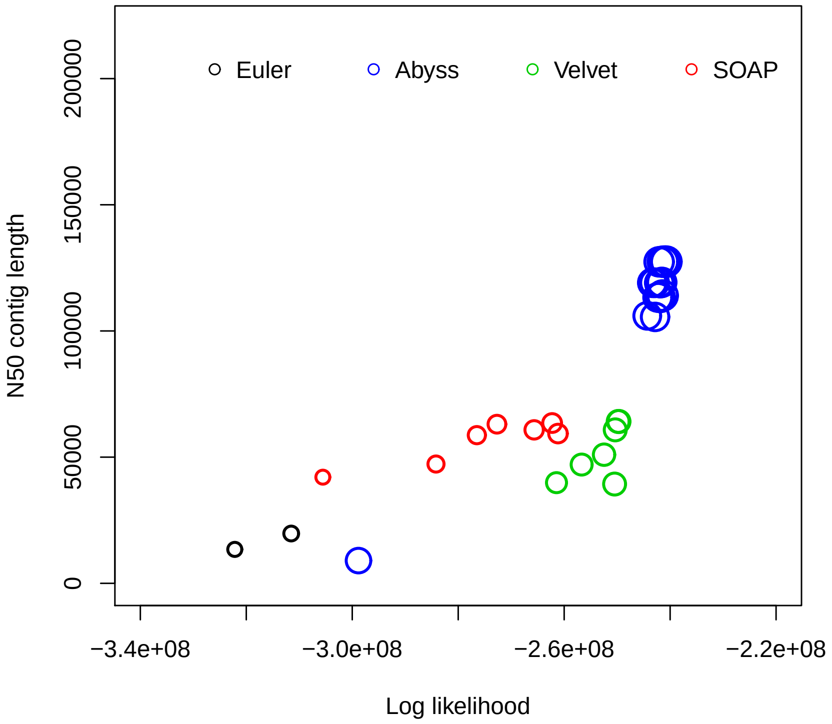

Figure S1: Log likelihood vs N50 contig length for *E. coli*. Log likelihoods are shown on the x-axis and N50 contig lengths are shown on the y-axis. Each circle corresponds to an assembly generated using an assembler for some hash length and sizes of circles correspond to similarity with reference. The R^2 values are (i) log likelihood vs similarity: 0.9372048, (ii) log likelihood vs N50 contig length: 0.3316912, (iii) N50 contig length vs similarity: 0.4584904

Supplementary information for E. coli data from CLC bio

- Organism: *Escherichia coli*
- Total reads: 2609307
- Reads mapped using: BFAST-0.6.5a

Reference - provided

- Identifier: [NCBI: NC_010473.1]
- Likelihood: -69204665.425366
- Number of reads mapped: 2523195

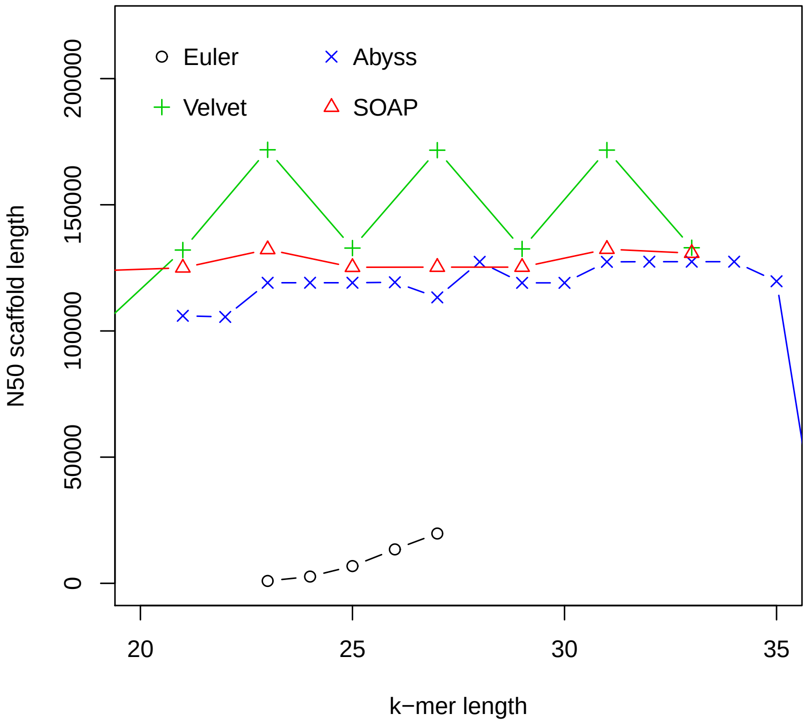

Figure S2: Hash length vs N50 scaffold length for *E. coli*

- Total length: 4686137 bases

Reference - conjectured

- Identifier: [NCBI: NC_000913.2]
- Likelihood: -57857459.428822
- Number of reads mapped: 2591992
- Total length: 4639675 bases

Assemblies

k-mer	# contigs	Log likelihood	# reads mapped	Total length	N50 scaffold	N50 contig	Diff
21	505	-59.21×10^6	2579014	4696557	82879	82879	40186
22	445	-58.99×10^6	2581729	4679494	95869	95869	34079
23	418	-59.16×10^6	2581745	4679963	92870	92870	35167
24	375	-58.98×10^6	2582234	4654094	89001	88061	34348
25	367	-58.94×10^6	2582400	4768360	88276	88276	34030
26	362	-59.16×10^6	2583036	4756869	78513	78513	31967
27	370	-58.85×10^6	2581930	4691177	80741	72160	36373
28	386	-59.38×10^6	2578904	4926998	59949	59185	34738
29	465	-60.05×10^6	2571176	4839950	42041	33858	40728
30	914	-63.74×10^6	2533423	4850165	16657	13990	47853

Table S5: Details of ABySS assemblies of *E. coli* from CLC bio

k-mer	# contigs	Log likelihood	# reads mapped	Total length	N50 scaffold	N50 contig	Diff
21	620	-65.65×10^6	2522602	4692994	32728	30483	98983
22	569	-64.66×10^6	2528669	4691836	33655	29444	93766
23	527	-64.34×10^6	2527001	4598511	31478	27051	93579
24	560	-65.73×10^6	2517944	4608041	33556	22946	102399
25	667	-68.01×10^6	2494175	4561288	27187	14191	124413
26	830	-73.72×10^6	2431998	4545734	19849	8495	175362
27	1131	-84.04×10^6	2312331	4473173	12353	4780	280823

Table S6: Details of Euler-sr assemblies of *E. coli* from CLC bio

k-mer	# contigs	Log likelihood	# reads mapped	Total length	N50 scaffold	N50 contig	Diff
21	180	-64.41×10^6	2530857	4538915	82523	53402	117331
23	176	-63.66×10^6	2536802	4541478	94647	57729	113126
25	179	-63.93×10^6	2538168	4544165	94659	58755	112015
27	175	-64.23×10^6	2527604	4539301	95159	46483	121115
29	200	-72.31×10^6	2438802	4488341	83775	12597	203884

Table S7: Details of SOAPdenovo assemblies of *E. coli* from CLC bio

k-mer	# contigs	Log likelihood	# reads mapped	Total length	N50 scaffold	N50 contig	Diff
19	2091	-82.01×10^6	2352836	4532537	4112	4112	128441
21	198	-62.95×10^6	2542960	4542798	78546	53459	100298
23	896	-71.06×10^6	2471662	4532134	11286	11286	113920
25	1075	-71.52×10^6	2459554	4542157	8352	8386	111040

Table S8: Details of Velvet assemblies of *E. coli* from CLC bio

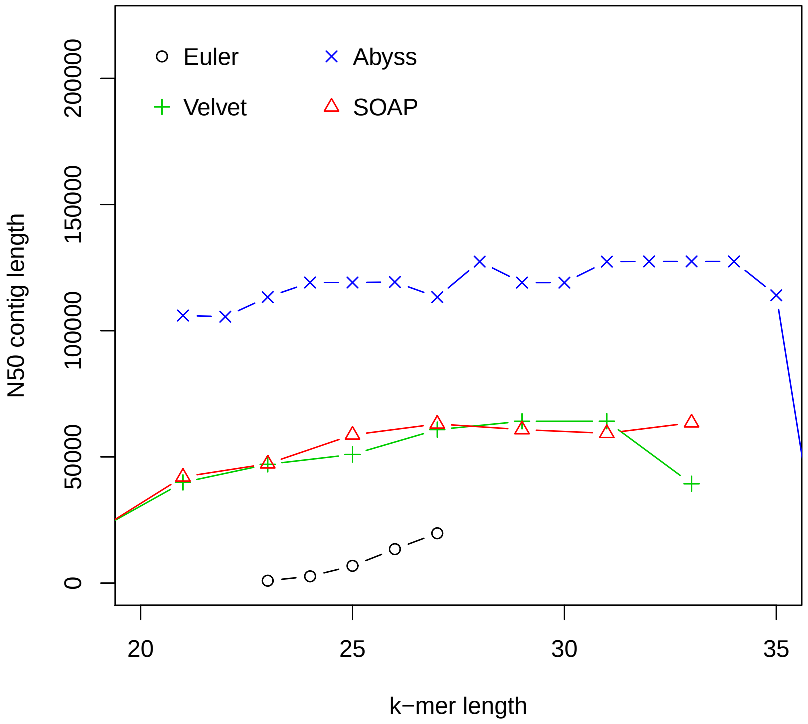

Figure S3: Hash length vs N50 contig length for *E. coli*

Supplementary information for G. clavigera data

- Organism: *Grosmannia clavigera*
- Total reads: 41034877
- Reads mapped using: BFAST-0.6.5a

References

- Identifier: [DDBJ/EMBL/GenBank: ACXQ00000000]
- Likelihood: -1879322697.243536
- Number of reads mapped: 35708831
- Total length: 29128742 bases

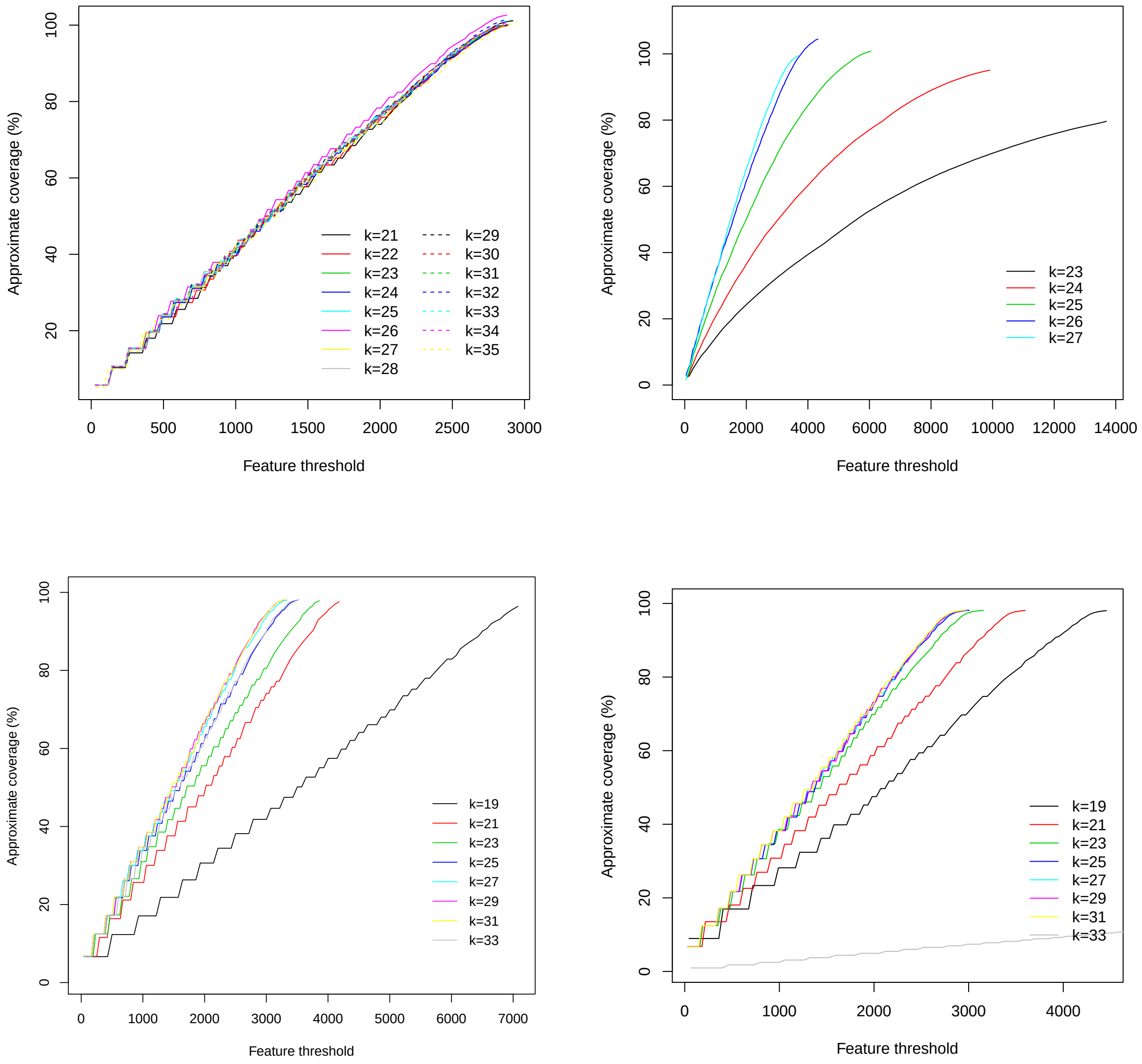

Figure S4: Feature response curves for vs (a) ABySS, (b) Euler-sr, (c) SOAPdenovo, (d) Velvet assemblies of *E. coli*

- Identifier: [DDBJ/EMBL/GenBank: ACYC00000000]
- Likelihood: -1770350912.163320
- Number of reads mapped: 36287407
- Total length: 29518845 bases

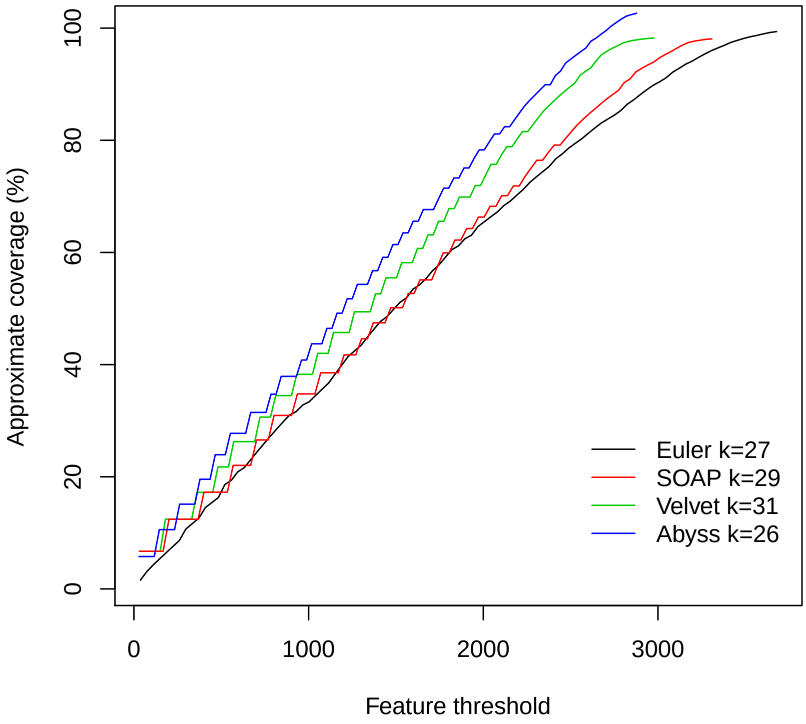

Figure S5: Feature response curves for assemblies of *E. coli* by differenct assemblers

Assemblies

k-mer	# contigs	Log likelihood	# reads mapped	Total length	N50 scaffold	N50 contig	Diff
21	256124	-21.38×10^8	32979149	37142184	17220	16671	2809452
22	232501	-	-	36709685	27945	25559	2700584
23	210071	-20.71×10^8	33306434	36657119	37813	35026	2594137
24	192943	-	-	35750082	55105	49172	2482608
25	184177	-20.41×10^8	33472590	36531590	62982	58787	2382831
26	170997	-	-	37378311	77784	67029	2267368
27	159554	-20.23×10^8	33633432	35707870	93477	82886	2149608
28	149309	-	-	35786179	98522	92594	2028791
29	141391	-19.78×10^8	33809082	34799760	106367	92588	1906317
30	128886	-	-	34297703	112410	98015	1763278
31	118223	-19.40×10^8	34087970	34330058	119303	106618	1621203
32	107767	-	-	34689640	130674	105063	1484476
33	96232	-19.32×10^8	34385760	34434911	132294	113136	1266955
34	87415	-	-	35763495	135634	116906	1150820
35	77636	-19.12×10^8	34618713	34532607	135175	115835	1067088
36	69079	-	-	34687680	132362	104995	992028
37	61086	-18.96×10^8	34821797	34949964	136072	102040	922206
38	52639	-	-	34591823	118013	85684	886238
39	46557	-18.96×10^8	35034414	34312824	101526	63060	873592
40	42136	-	-	32248177	54066	28644	976543

Table S9: Details of ABySS assemblies of *G. clavigera*. '-' indicates likelihood not computed

k-mer	# contigs	Log likelihood	# reads mapped	Total length	N50 scaffold	N50 contig	Diff
23	718	-21.94×10^8	33595856	28168246	404931	9519	3020692
25	854	-21.78×10^8	33889880	28382967	471227	16994	2837023
27	1077	-21.26×10^8	33906837	28633019	424982	24420	2679202
29	1319	-21.18×10^8	33943297	29022797	472893	28732	2520229
31	1672	-20.50×10^8	34161562	29315722	357348	31279	2362441
33	1959	-19.99×10^8	34537448	29386051	363057	26110	2227202
35	2319	-19.59×10^8	34962836	29343677	329057	15974	2166290
37	2737	-18.77×10^8	35088054	29073407	328162	4779	2527367
39	4411	-22.52×10^8	33276354	29618592	30833	440	7574440

Table S10: Details of SOAPdenovo assemblies of *G. clavigera*

k-mer	# contigs	Log likelihood	# reads mapped	Total length	N50 scaffold	N50 contig	Diff
21	4591	-21.81×10^8	33369710	26845917	21877	10000	3088905
23	1250	-21.11×10^8	34259414	26755488	174576	17299	2960501
25	1247	-19.86×10^8	34626195	26820775	256321	22175	2870534
27	1513	-19.26×10^8	34846513	26974390	313211	31692	2707470
29	1703	-19.17×10^8	34886953	27199402	322108	38029	2545990
31	1967	-19.07×10^8	34984676	27514303	304883	41579	2339277
33	2046	-19.00×10^8	35210381	27736636	332958	39448	2129331
35	2066	-18.79×10^8	35394715	28019566	322296	28230	1876746
37	2064	-18.87×10^8	35469983	28278498	279463	12808	1794095
39	2164	-18.94×10^8	35395912	28837034	181012	3210	2220725

Table S11: Details of Velvet assemblies of *G. clavigera*

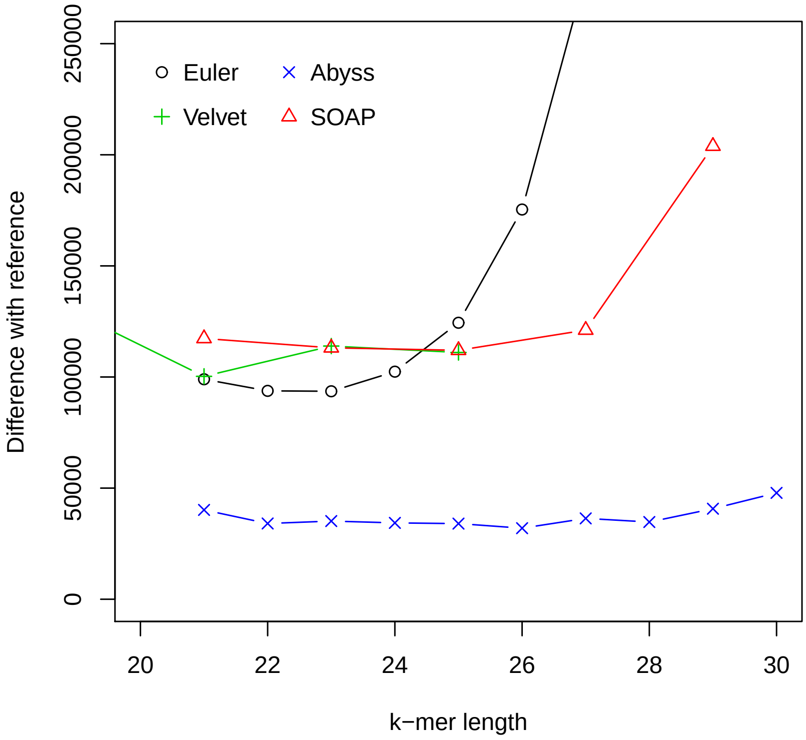

Figure S6: Hash length vs difference from reference for CLC bio *E. coli* data. Differences between assemblies and the reference are shown on the y-axis where difference refers to numbers of bases in the reference not covered by the assembly or are different in the reference and the assembly.

Supplementary information for GAGE assemblies

- Reads mapped using Bowtie 2 - 2.0.0 - beta 6

	Library 1		Library 2	
Assembler	Likelihood	# reads mapped	Likelihood	# reads mapped
ABySS	-63.67×10^6	530004	-16.97×10^7	706226
ALLPATHS-LG	-80.69×10^6	497645	-16.46×10^7	722683
Bambus2	-74.04×10^6	497912	-16.36×10^7	702615
MSR-CA	-92.54×10^6	475548	-16.59×10^7	716453
SGA	-79.25×10^6	483150	-18.68×10^7	535786
SOAPdenovo	-64.69×10^6	518963	-17.08×10^7	693421
Velvet	-65.18×10^6	515646	-16.76×10^7	688261
Reference	-63.55×10^6	530582	-16.03×10^7	738136

Table S12: Likelihoods of GAGE assemblies of *S. aureus*

	Library 1		Library 2	
Assembler	Likelihood	# reads mapped	Likelihood	# reads mapped
ABySS	-11.18×10^7	830206	-16.37×10^7	368991
ALLPATHS-LG	-11.25×10^7	840079	-15.35×10^7	397859
Bambus2	-13.64×10^7	782413	-18.92×10^7	329183
CABOG	-16.17×10^7	735552	-23.06×10^7	287180
MSR-CA	-13.74×10^7	786563	-17.87×10^7	368515
SGA	-12.06×10^7	776980	-19.52×10^7	254567
SOAPdenovo	-11.23×10^7	832984	-16.44×10^7	379975
Velvet	-11.47×10^7	825280	-17.29×10^7	350845
Reference	-11.04×10^7	844992	-14.95×10^7	410758

Table S13: Likelihoods of GAGE assemblies of *R. sphaeroides*

	Library 1		Library 2	
Assembler	Likelihood	# reads mapped	Likelihood	# reads mapped
ABySS	-82.28×10^7	17668025	-15.21×10^8	4428441
ALLPATHS-LG	-96.97×10^7	17409906	-13.11×10^8	5712663
Bambus2	-22.77×10^8	14162003	-	-
CABOG	-90.14×10^7	17579185	-12.25×10^8	5854239
MSR-CA	-10.44×10^8	16817275	-	-
SGA	-10.68×10^8	16065518	-	-
SOAPdenovo	*	*	-	-
Velvet	-13.07×10^8	14382698	-	-
Reference	-77.90×10^7	17938368	-11.25×10^8	6039649

Table S14: Likelihoods of GAGE assemblies of human chromosome 14. * Likelihood not computed as reads could not be mapped with Bowtie 2

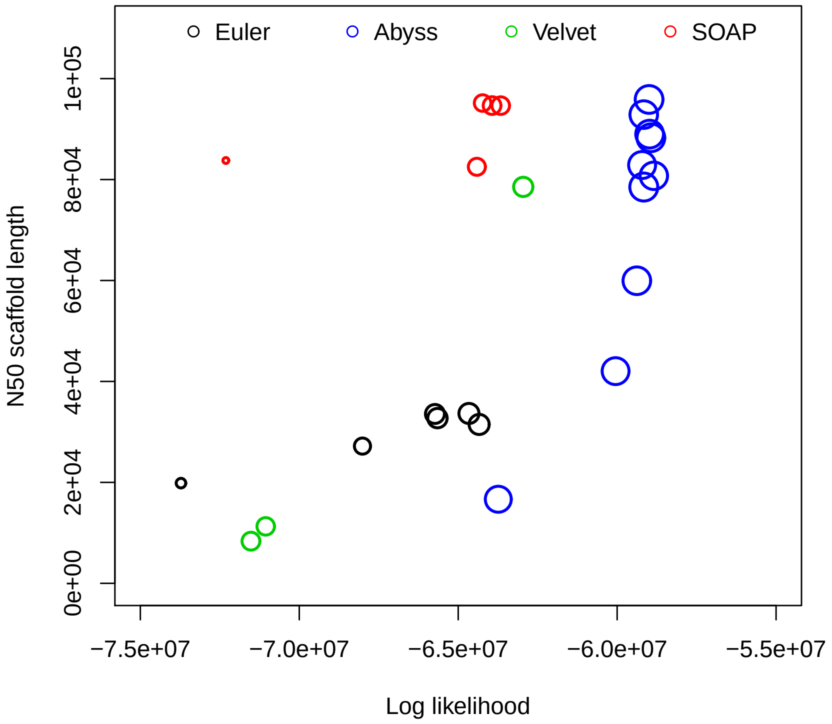

Figure S7: Log likelihood vs N50 scaffold length for CLC bio *E. coli* data. Log likelihoods are shown on the x-axis and N50 scaffold lengths are shown on the y-axis. Each circle corresponds to an assembly generated using an assembler for some hash length and sizes of circles correspond to similarity with reference. The R^2 values are (i) log likelihood vs similarity: 0.7508, (ii) log likelihood vs N50 scaffold length: 0.4502, (iii) N50 scaffold length vs similarity: 0.1525

Supplementary information for Assemblathon 1 data

- Reads mapped using BFAST-0.7.0a
- Due to an issue with mapping first 21200000, 10550000, 10550000 reads from libraries of insert size 200, 3000, 10000 respectively were mapped

Assembler	Likelihood	#reads mapped	Coverage
ASTR 1	-15.73×10^8	19583345	90.9
WTSI-P 1	-84.10×10^7	21111310	98.7
WTSI-P 2	-83.38×10^7	21140835	98.6
EBI 1	-91.35×10^7	20903073	97.7
EBI 2	-12.50×10^8	19866346	92.9
WTSI-S 1	-88.44×10^7	21021575	97.8
WTSI-S 2	-88.12×10^7	21026735	97.8
WTSI-S 3	-88.56×10^7	21008685	97.8
WTSI-S 4	-83.84×10^7	21111281	98.3
CRACS 1	-10.30×10^8	20701940	96.3
CRACS 2	-10.68×10^8	20616519	95.9
CRACS 3	-11.02×10^8	20588439	95.6
BCCGSC 1	-82.38×10^7	21140990	98.6
BCCGSC 2	-82.36×10^7	21142978	98.6
BCCGSC 3	-82.25×10^7	21143989	98.7
BCCGSC 4	-82.23×10^7	21148816	98.7
BCCGSC 5	-82.20×10^7	21148400	98.7
DOEJGI 1	-86.84×10^7	21098465	97.3
IRISA 1	-11.46×10^8	20504763	93.7
IRISA 2	-12.42×10^8	20193993	92.8
IRISA 3	-11.99×10^8	20349895	92.9
IRISA 4	-10.12×10^8	20762461	95.7
IRISA 5	-10.97×10^8	20563254	94.6
CSHL 1	-18.69×10^8	18879773	87.2
CSHL 2	-83.01×10^7	21140814	98.5
DCSISU 1	-11.31×10^8	20525628	94.3
IoBUGA 1	-85.73×10^7	21106253	98.3
IoBUGA 2	-85.69×10^7	21103249	98.3
IoBUGA 3	-87.99×10^7	21057492	98.1
UCSF 1	-21.98×10^8	18216040	83.7
RHUL 1	-82.92×10^7	21138821	98.5
RHUL 2	-83.03×10^7	21140544	98.5
RHUL 3	-81.56×10^7	21142931	98.7
RHUL 4	-96.40×10^7	20868381	97.0
RHUL 5	-82.61×10^7	21148936	98.7
GACWT 1	-18.32×10^8	18734425	87.6
GACWT 2	-25.98×10^8	17023161	79.5
GACWT 3	-19.33×10^8	18455366	86.4
CIUoC	-27.38×10^8	17087357	78.5
BGI 1	-84.13×10^7	21108642	98.8
Broad 1	-92.49×10^7	21026955	98.3

Table S15: Assemblathon 1 likelihoods

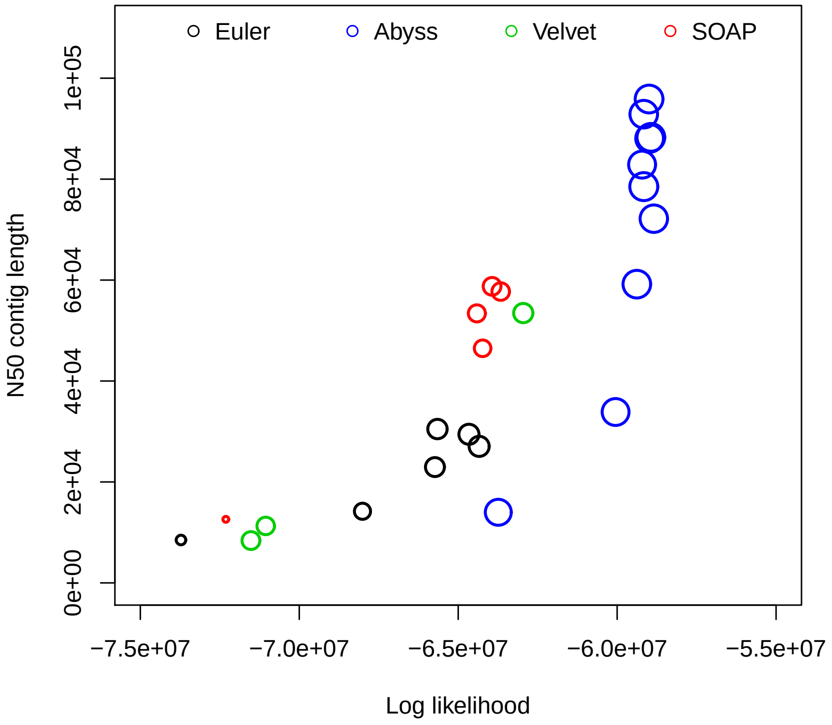

Figure S8: Log likelihood vs N50 contig length for CLC bio *E. coli* data. Log likelihoods are shown on the x-axis and N50 contig lengths are shown on the y-axis. Each circle corresponds to an assembly generated using an assembler for some hash length and sizes of circles correspond to similarity with reference. The R^2 values are (i) log likelihood vs similarity: 0.7508, (ii) log likelihood vs N50 contig length: 0.6456, (iii) N50 contig length vs similarity: 0.4867

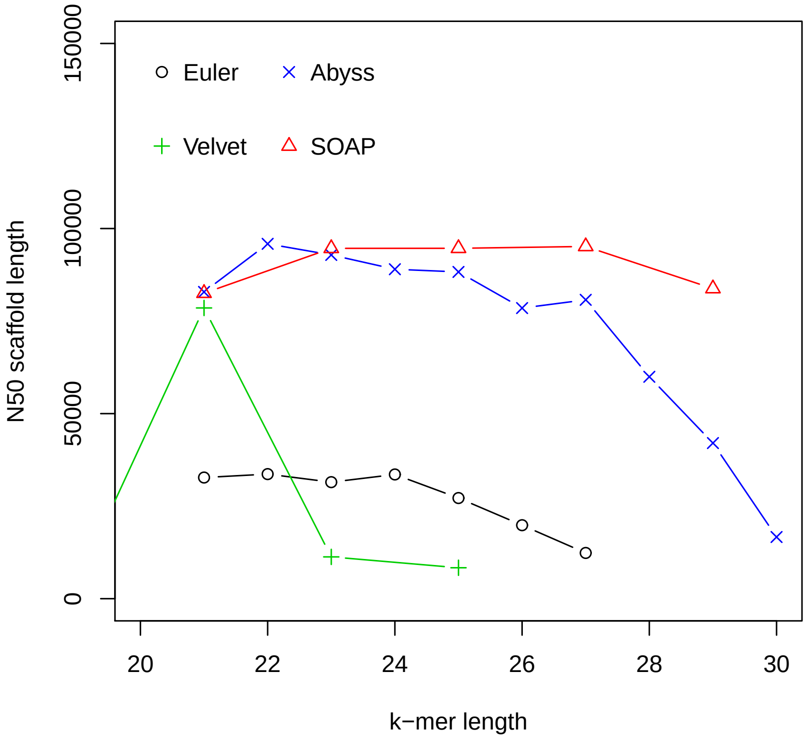

Figure S9: Hash length vs N50 scaffold length for CLC bio *E. coli* data

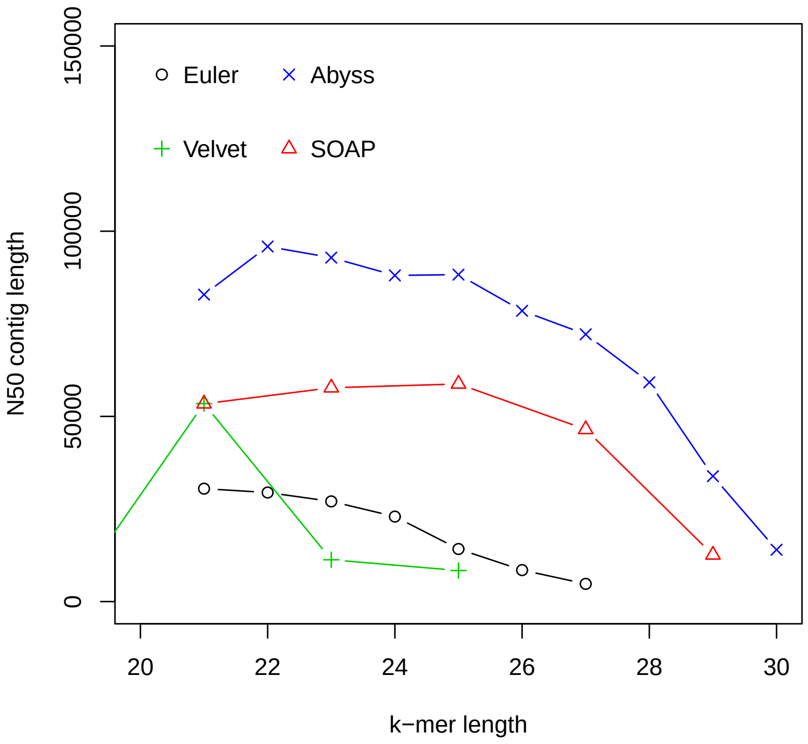

Figure S10: Hash length vs N50 contig length for CLC bio *E. coli* data

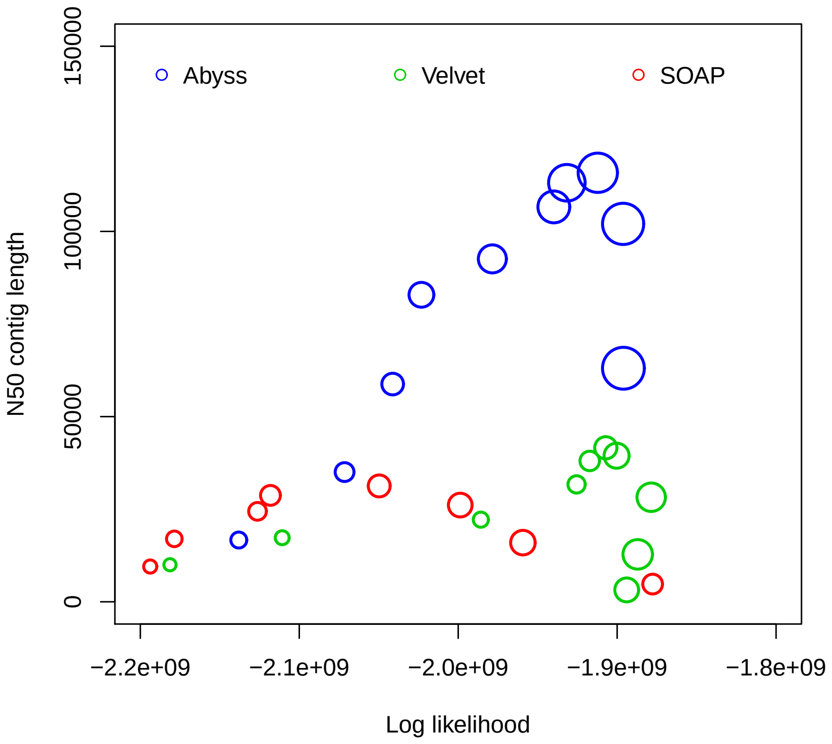

Figure S11: Log likelihood vs N50 contig length for *G. clavigera.* Log likelihoods are shown on the x-axis and N50 contig lengths are shown on the y-axis. Each circle corresponds to an assembly generated using an assembler for some hash length and sizes of circles correspond to similarity with reference. The R^2 values are (i) log likelihood vs similarity: 0.4545793, (ii) log likelihood vs N50 contig length: 0.1582344, (iii) N50 contig length vs similarity: 0.3287432

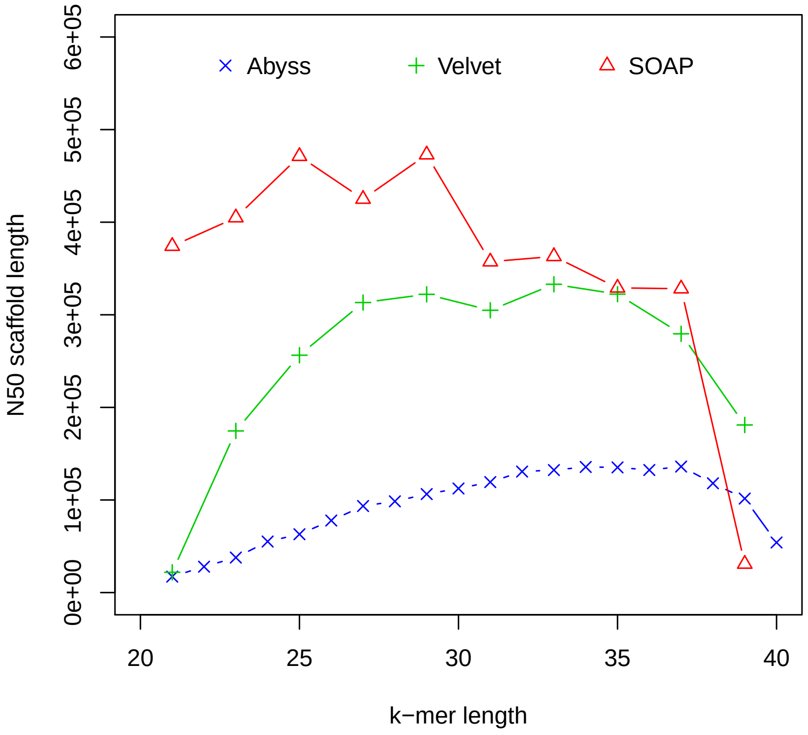

Figure S12: Hash length vs N50 scaffold length for *G. clavigera*

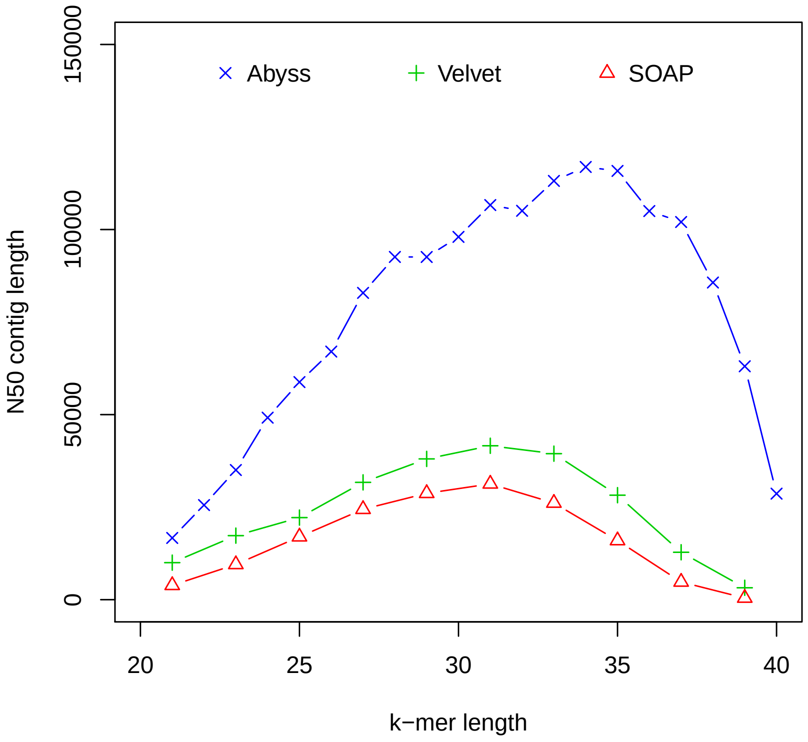

Figure S13: Hash length vs N50 contig length for *G. clavigera*

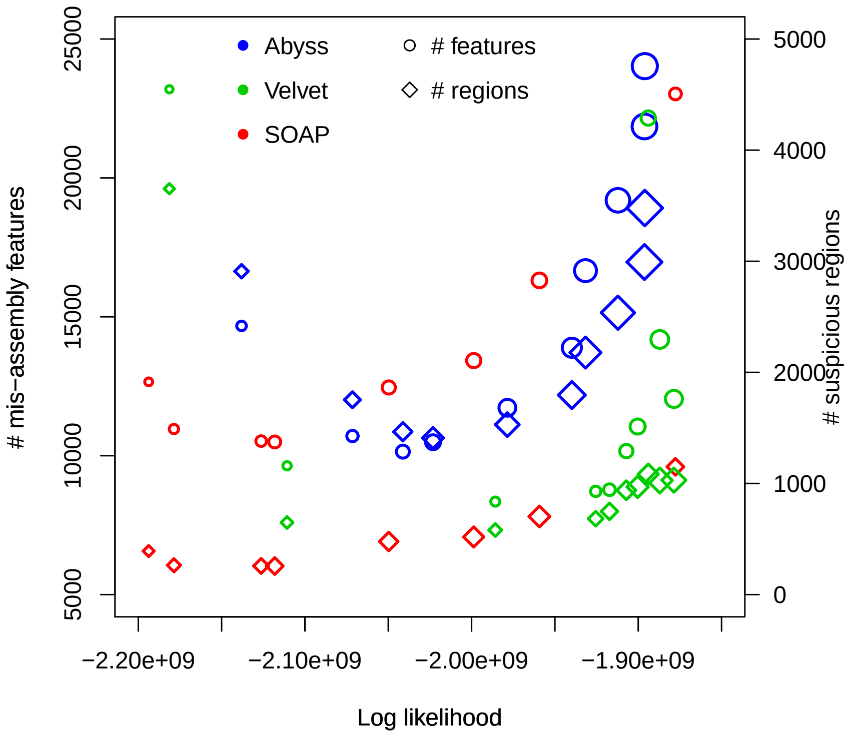

Figure S14: Log likelihood vs Numbers of mis-assembly features and suspicious regions for *G. clavigera* Log likelihoods are shown on the x-axis and Numbers of mis-assembly features and suspicious regions reported by amosvalidate are shown on the y-axis. Each symbol corresponds to an assembly generated using an assembler for some hash length and sizes of symbols correspond to similarity with reference. The R^2 values are (i) log likelihood vs # mis-assembly features: 0.0726, (ii) log likelihood vs # suspicious regions: 0.0002, (iii) similarity vs # mis-assembly features: 0.2429, (iv) similarity vs # suspicious regions: 0.0588

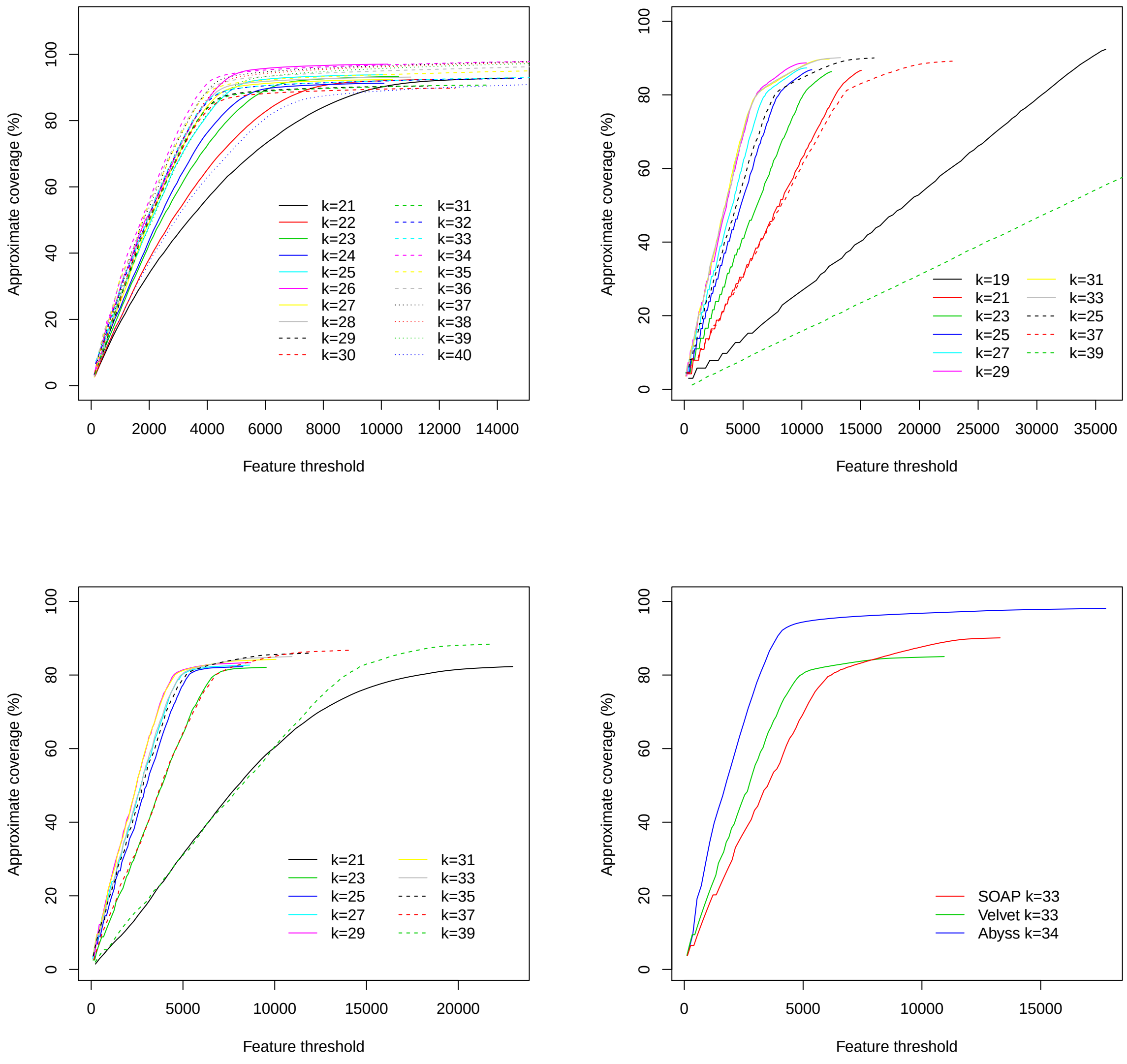

Figure S15: Feature response curves for vs (a) ABySS, (b) SOAPdenovo, (c) Velvet, (d) all assemblies of *G. clavigera*
